# Study on Environmental Causes and SNPs of MTHFR, MS and CBS Genes Related to Congenital Heart Disease

**DOI:** 10.1371/journal.pone.0128646

**Published:** 2015-06-02

**Authors:** Hui Shi, Shiwei Yang, Yan Liu, Peng Huang, Ning Lin, Xiaoru Sun, Rongbin Yu, Yuanyuan Zhang, Yuming Qin, Lijuan Wang

**Affiliations:** 1 Jiangsu Institute of Planned Parenthood Research, Nanjing, Jiangsu, 210036, China; 2 Department of Cardiology, Nanjing Children's Hospital, Nanjing Medical University, Nanjing, 210008, China; 3 Nanjing Medical University, Nanjing, Jiangsu, 210029, China; University of Louisville, UNITED STATES

## Abstract

**Purpose:**

Congenital heart diseases (CHD) are among the most common birth defects in China. Environmental causes and folate metabolism changes may alter susceptibility to CHD. The aim of this study is to evaluate the relevant risk-factors of children with CHD and their mothers.

**Methods:**

138 children with CHD and 207 normal children for controls were recruited. Their mothers were also enlisted in this study and interviewed following a questionnaire about their pregnant history and early pregnancy situation. Five single nucleotide polymorphisms (SNPs) in methylenetetrahydrofolate reductase (*MTHFR*), methionine synthase (*MS*) and cystathionine β-synthase (*CBS*) of mothers and children were genotyped.

**Results:**

There were significant differences in the gender of children, occupation of mothers, family history with CHD, history of abortion, history of adverse pregnancy, early pregnancy health, fetus during pregnancy, pesticide exposure and drug exposure in CHD group and control group ( *P* < 0.05). Logistic regression analyses showed that after adjustment for above factors, *MTHFR* rs1801131 were significantly associated with their offspring CHD risk in mothers. Compared with the mothers whose MTHFR were rs1801131 AA and AC genotypes, the mothers who got a mutation of MTHFR rs1801131 CC genotypes had a 267% increase in risk of given birth of a CHD children (OR=3.67,95%CI=1.12-12.05). Meanwhile, *MTHFR* rs1801131 were significantly associated with CHD susceptibility in children (OR = 1.42, 95% CI = 1.00-2.44 in additive model).

**Conclusions:**

Besides mothers’ social and fertility characteristics, our results suggested that the genetic variants in folate metabolism pathway might be one of the most related risk-factors of CHD. *MTHFR* rs1801131 were identified as loci in Chinese population that were involved in CHD.

## Introduction

Congenital heart diseases (CHDs) have been the highest incidence of birth defects and increased yearly in China since 2005. It affected 43.22 per 10 thousand live births in 2013 [1]. The CHDs are multifactorial disease and their etiology is not fully understood. The majority types of CHDs are proved to be affected by both genetic and environmental factors [2]. Some reports showed the association between the use of multivitamin and CHDs risk which could be reduced 40%-60% by pre-conceptional multivitamin use [3,4].

There is evidence suggesting that polymorphisms in folate metabolism could alter susceptibility to CHDs. Thus, there are plenty studies on SNPs of the genes involved in the folate metabolism pathway, especially the 3 major enzymes including methylenetetrahydrofolate reductase (*MTHFR*), methionine synthase (*MS*) and cystathionine β-synthase (*CBS*). However, these studies brought about controversial results [5–8]. The objective of the present study was to evaluate the prevalence estimates of environment causes and the investigation of the association between the genotype and the disease. The polymorphisms rs1801131 and rs1801133 in *MTHFR* gene, rs1805087 in *MS* gene and rs2124459, rs2850144 in *CBS* gene were detected in children with CHD and their mothers and compared with which of the control group in Jiangsu Province, China.

## Materials and Methods

### Ethics Statement

The study had been approved by the Ethics Review Committee of the Jiangsu Institute of Planned Parenthood Research, Nanjing, China. Prior written informed consent was obtained from all the adult participants and the guardians on behalf of the children enrolled in the study.

### Participants

From May, 2012 to December, 2013, all consenting children participants in the study were recruited from the Nanjing Children’s Hospital, Jiangsu Province, China. Their mothers were interviewed face-to-face to collect demographic data and exposure information, such as child-bearing age, history of abortion, history of adverse pregnancy, cigarette smoking, and alcohol drinking etc. Each participant donated 3 mL venous blood for serological tests and host DNA genotyping. If the frequency of variant genotypes is 30%, odds ratio is 2.0, *α* is 0.05 and *β* is 0.2, the desired sample size is 147.

#### DNA collection and genotyping

Genomic DNA was extracted from a leukocyte pellet by traditional proteinase K digestion and followed by phenol-chloroform extraction and ethanol precipitation. All SNPs (*MTHFR* rs1801131 and rs1801133, *MS* rs1805087, *CBS* rs2124459 and rs2850144) were genotyped by the Taq-Man allelic discrimination assay on an ABI 7900 system (Applied Biosystems, La Jolla CA). The information on primers and probes are shown in S1 Table. All the genotyping assays was performed without knowing the subjects’ case and control status; two blank (i.e., water) controls in each 384-well format were used for quality control, and more than 10% of samples were randomly selected to repeat, yielding a 99% concordant. The success rates of genotyping for these polymorphisms were all above 98%.

#### Statistical analysis

Differences in the general demographic characteristics were calculated by the Student t test or One-Way ANOVA and the chi-square (χ2) test. Odds ratios (ORs) and their 95% confidential intervals (CIs) were calculated as a measure of difference in the response rate using logistic regression analysis. The adjustment factors included gender of children, occupation of the mothers, family history with CHD, history of abortion, history of adverse pregnancy, early pregnancy health, and fetus during pregnancy, pesticide exposure and drug exposure. The haplotype frequencies based on the observed genotypes were estimated by PHASE software (v2.1). The statistical analyses were performed using Statistical Analysis System software (version 9.1.3, SAS Institute, Cary, NC). All *P* < 0.05 in a two-sided test was considered statistically significant.

## Results

In this study, a total of 345 children and 369 mothers with available blood sample were included. The CHD group recruited 137 mothers and their children. Another 16 mothers and 1 child were included without matching. The control group contained 207 mothers and their children and another 9 mothers without matching children. The selected characteristics of the mothers in CHD group and the controls in the study were shown in Table 1. There were significant differences in the gender of children, occupation of the mothers, family history with CHD, history of abortion, history of adverse pregnancy, early pregnancy health, fetus during pregnancy, pesticide exposure and drug exposure in two groups (*P* < 0.05 for both comparisons).

**Table 1:** Demographic and selected variables in mothers of CHD children and controls.

Variables	Control N(%)	Case N(%)	*P*
**Gender of children**			
Male	124(57.41)	61(38.85)	0.001
Female	92(42.59)	96(61.15)	
**Child-bearing age**			
Age, year (mean ± SD)	25.39±3.28	26.16±4.69	0.083
<25	77(44.51)	75(48.70)	0.505
≥25	96(55.49)	79(51.30)	
**Education**			
Middle school and lower	122(70.11)	95(60.51)	0.082
High school and above	52(29.89)	62(39.49)	
**Occupation**			<0.001
Farmer	92(51.99)	34(21.66)	
Worker	27(15.25)	36(22.93)	
Server	4(2.26)	17(10.83)	
Businessman	14(7.91)	12(7.64)	
Government worker	3(1.69)	3(1.91)	
Others	37(20.90)	55(35.03)	
**Family history with CHD**			
No	174(100)	149(94.90)	<0.001
Yes	0	8(5.10)	
**History of abortion**			<0.001
No	152(87.36	92(54.62)	
Yes	22(12.64)	65(45.38)	
**history of adverse pregnancy**			<0.001
No	160(91.95)	96(61.15)	
Yes	14(8.05)	61(38.85)	
**Early pregnancy health**			<0.001
Healthy	158(90.80)	78(49.68)	
Illness	16(9.20)	79(50.32)	
**Fetus during pregnancy**			0.028
Normal	172(98.85)	147(94.23)	
Abnormal	2(1.15)	9(5.77)	
**Pesticide exposure**			0.008
No	173(99.42)	148(94.27)	
Yes	1(0.58)	9(5.73)	
**Drug exposure**			<0.001
No	168(96.55)	116(73.89)	
Yes	6(3.45)	41(26.11)	

Abbreviation: SD, standard deviation.

The genotype percentage values for five SNPs (*MTHFR* rs1801131 and rs1801133, *MS* rs1805087, *CBS* rs2124459 and rs2850144) have been given in Table 2 for the mothers. All the distributions were found to be in agreement with Hardy-Weinberg equilibrium. Logistic regression analyses showed that *MTHFR* rs1801131, *CBS* rs2124459 and rs2850144 were significantly associated with CHD susceptibility in mothers. However, after adjustment for gender of children, occupation of mothers, family history with CHD, history of abortion, history of adverse pregnancy, early pregnancy health, fetus during pregnancy, pesticide exposure and drug exposure, only rs1801131 (A *vs*. C) variant genotypes significantly increased their offspring CHD risk, when compared with control group of mothers (OR = 3.67, 95% CI = 1.12–12.05 in Recessive model) (Table 2).

**Table 2:** Association of selected SNPs with in CHD in mothers.

Genotype	Control N(%)	Case N(%)	OR (95% CI)	*P* value	AOR (95% CI)	*P* value
rs1801133						
CC	70(32.41)	55(35.95)	1		1	
CT	101(46.76)	68(44.44)	0.86(0.54–1.37)	0.518	0.68(0.36–1.31)	0.253
TT	45(20.83)	30(19.61)	0.85(0.47–1.52)	0.580	0.79(0.35–1.76)	0.561
Dominant*			0.84(0.54–1.30)	0.429	0.70(0.38–1.28)	0.245
Recessive*			0.92(0.55–1.54)	0.750	0.96(0.47–1.97)	0.912
Additive*			0.91(0.69–1.22)	0.533	0.86(0.56–1.28)	0.451
rs1801131						
AA	157(72.68)	95(62.09)	1		1	
AC	53(24.54)	39(25.49)	1.22(0.75–1.97)	0.210	0.71(0.34–1.47)	0.357
CC	6(2.78)	19(12.42)	**5.23(2.02–13.57)**	**0.001**	**3.46(1.02–11.35)**	**0.046**
Dominant			**1.61(1.03–2.50)**	**0.035**	1.03(0.54–1.95)	0.934
Recessive			**4.93(1.92–12.65)**	**0.001**	**3.67(1.12–12.05)**	**0.032**
Additive			**1.72(1.22–2.42)**	**0.002**	1.28(0.80–2.06)	0.304
rs2124459						
CC	124(57.41)	103(67.32)	1		1	
CT	69(31.94)	40(26.14)	0.70(0.44–1.12)	0.133	0.90(0.29–1.32)	0.757
TT	23(10.65)	10(6.54)	0.52(0.24–1.15)	0.107	0.36(0.10–1.28)	0.114
Dominant			**0.65(0.42–0.99)**	**0.049**	0.74(0.40–1.37)	0.336
Recessive			0.58(0.27–1.26)	0.171	0.37(0.10–1.29)	0.118
Additive			**0.71(0.51–0.99)**	**0.044**	0.55(0.45–1.15)	0.172
rs1805087						
AA	176(81.48)	122(79.74)	1		1	
AG	37(17.13)	30(19.61)	1.17(0.69–1.99)	0.565	1.05(0.48–2.30)	0.894
GG	3(1.39)	1(0.65)	0.48(0.05–4.68)	0.528	0.79(0.04–14.10)	0.871
Dominant			1.11(0.66–1.87)	0.698	1.03(0.48–2.20)	0.942
Recessive			0.46(0.06–4.57)	0.508	0.77(0.05–13.92)	0.857
Additive			1.06(0.65–1.71)	0.824	1.01(0.51–2.03)	0.968
rs2850144						
CC	145(67.13)	86(56.21)	1		1	
CG	55(25.46)	50(32.68)	1.54(0.96–2.44)	0.073	1.65(0.84–3.22)	0.178
GG	16(7.41)	17(11.11)	1.79(0.86–3.72)	0.119	1.90(0.98–7.81)	0.095
Dominant			**1.57(1.03–2.41)**	**0.038**	1.88(0.95–3.57)	0.071
Recessive			1.55(0.76–3.18)	0.230	2.45(0.77–5.81)	0.148
Additive			**1.40(1.02–1.93)**	**0.035**	1.69(0.98–2.55)	0.067

Logistic regression analyses adjusted for gender of children, occupation of mothers, family history with CHD, history of abortion, history of adverse pregnancy, early pregnancy health, fetus during pregnancy, pesticide exposure and drug exposure.

*Dominant means wild vs. heterozygous+ homozygous, recessive means wild+ heterozygous vs. homozygous, additive means wild vs. heterozygous vs. homozygous.

We then used hereditary analysis for the *MTHFR*, *MS* and *CBS* polymorphism to test the relationship between those five SNPs in 344 matching pair mother and their children (Table 3). It was observed that the five SNPs in mothers were significantly associated with the SNPs in their offspring (*P* < 0.001).

**Table 3:** Hereditary analysis for the MTHFR, MS and CBS polymorphism.

Mother	Offspring		*P*
**rs1801133**	**CC**	**CT /TT**	**<0.001**
**CC**	67(57.26)	50(42.74)	
**CT /TT**	50(22.03)	177(77.97)	
**rs1801131**	AA	AC/CC	**<0.001**
**AA**	188(78.66)	48(21.34)	
**AC /CC**	51(45.71)	57(54.29)	
**rs1805087**	AA	AG/GG	**<0.001**
**AA**	242(86.43)	34(53.13)	
**AG /GG**	38(13.57)	30(46.88)	
**rs2124459**	CC	CT/TT	**<0.001**
**CC**	142(74.74)	69(44.81)	
**CT /TT**	48(25.26)	85(55.19)	
**rs2850144**	CC	CG/GG	**<0.001**
**CC**	174(77.33)	49(41.18)	
**CG/GG**	51(22.67)	70(58.82)	

Table 4 showed the genotype percentage values for those five SNPs for the children. Logistic regression analyses showed that *MTHFR* rs1801131 and rs1801133 were significantly associated with CHD susceptibility. However, after adjustment for gender of children, occupation of mother, family history with CHD, history of abortion, history of adverse pregnancy, early pregnancy health, fetus during pregnancy, pesticide exposure and drug exposure, only rs1801131 (A *vs*. C) variant genotypes significantly increased host CHD risk, when compared with children without CHD (OR = 1.42, 95% CI = 1.00–2.44 in additive model) (Table 4).

**Table 4:** Association of selected SNPs with in CHD in children.

Genotype	Control N(%)	Case N(%)	OR (95% CI)	*P* value	AOR (95% CI)	*P* value
rs1801133 CC						
	80(38.65)	37(26.81)	1		1	
CT	85(40.06)	71(51.45)	**1.81(1.09–2.98)**	**0.021**	1.08(0.53–2.22)	0.826
TT	42(20.29)	30(21.74)	1.54(0.84–2.84)	0.162	1.60(0.69–3.70)	0.271
Dominant*			1.34(0.87–2.05)	0.186	0.96(0.53–1.74)	0.905
Recessive*			1.00(0.59–1.69)	0.993	1.29(0.63–2.66)	0.481
Additive*			1.28(0.95–1.73)	0.098	1.25(0.82–1.89)	0.299
rs1801131						
AA	163(80.20)	74 (53.63)	1		1	
AC	36(15.94)	57(41.30)	**3.87(2.34–6.44)**	**<0.001**	**2.04(1.02–4.08)**	**0.046**
CC	8(3.86)	7(5.07)	1.96(0.69–5.61)	0.208	1.50(0.44–5.19)	0.519
Dominant			**3.04(1.90–4.48)**	**<0.001**	**1.89(1.01–2.95)**	**0.047**
Recessive			1.24(0.44–3.49)	0.686	1.27(0.38–4.23)	0.697
Additive			**2.44(1.63–3.65)**	**<0.001**	**1.42(1.00–2.44)**	**0.049**
rs2124459						
CC	113(54.59)	78(56.53)	1		1	
CT	77(37.20)	46(33.33)	0.87(0.54–1.38)	0.543	1.49(0.75–2.94)	0.251
TT	17(8.21)	14(10.14)	1.19(0.56–2.56)	0.651	1.37(0.49–3.81)	0.549
Dominant			0.79(0.60–1.29)	0.371	1.53(0.79–2.97)	0.207
Recessive			1.17(0.56–2.45)	0.676	1.07(0.41–2.79)	0.898
Additive			0.99(0.72–1.39)	0.998	1.26(0.80–1.98)	0.316
rs1805087						
AA	174(84.06)	107(77.54)	1		1	
AG	33(15.94)	31(22.46)	1.53(0.88–2.64)	0.128	1.46(0.65–3.24)	0.358
GG	0	0	—		—	
Dominant			1.40(0.81–2.40)	0.225	1.25(0.58–2.72)	0.567
Recessive			—		—	
Additive			1.53(0.88–2.64)	0.128	1.46(0.65–3.24)	0.358
rs2850144						
CC	136(65.70)	90(65.23)	1		1	
CG	55(26.57)	34(24.63)	0.93(0.56–1.55)	0.791	1.44(0.72–2.89)	0.307
GG	16(7.73)	14(10.14)	1.32(0.62–2.84)	0.474	1.29(0.41–4.09)	0.667
Dominant			1.40(0.81–2.40)	0.225	1.25(0.58–2.72)	0.567
Recessive			1.25(0.59–2.64)	0.559	1.09(0.36–3.27)	0.877
Additive			1.07(0.71–1.49)	0.684	1.24(0.77–2.01)	0.378

Logistic regression analyses adjusted for gender of children, occupation of mothers, family history with CHD, history of abortion, history of adverse pregnancy, early pregnancy health, fetus during pregnancy, pesticide exposure and drug exposure.

*Dominant means wild vs. heterozygous+ homozygous, recessive means wild+ heterozygous vs. homozygous, additive means wild vs. heterozygous vs. homozygous.

Haplotype analyses for *MTHFR* gene were performed to define informative haplotypes associated with CHD risk. The results indicated that when compared with CA haplotype, the CC and TC haplotype suggested a risk effect in the mothers. However, compared to CA in the children, all other haplotypes containing variant alleles of the 2 SNPs were significantly associated with the risk of CHD (Table 5).

**Table 5:** Results of haplotype association analysis of CHD for *MTHFR*.

Haplotype	Controls	Cases	OR (95%CI)	*P*
Mothers				
CA	177 (41.0)	114 (37.3)	1.00	-
TA	190 (44.0)	115 (37.6)	0.94 (0.68–1.31)	0.712
CC	64 (14.8)	64 (20.9)	1.55 (1.02–2.36)	0.040
TC	1 (0.2)	13 (4.2)	20.18 (2.60–156.40)	0.004
Children				
CA	199 (48.1)	84 (30.5)	1.00	—
TA	166 (40.1)	121 (43.8)	1.73 (1.22–2.44)	0.002
CC	46 (11.1)	61 (22.1)	3.14 (1.98–4.98)	< 0.001
TC	3 (0.7)	10 (3.6)	7.89 (2.12–29.42)	0.002

Logistic regression analyses adjusted for gender of children, occupation of mothers, family history with CHD, history of abortion, history of adverse pregnancy, early pregnancy health, fetus during pregnancy, pesticide exposure and drug exposure.

## Discussion

CHDs are proved to be affected by both genetic and environmental factors which from biology, chemistry, physics, social psychology and others. Our previous meta analysis of risk factors of CHD of Chinese perinatal children revealed that spontaneous abortion, fetal abnormality, medicine use in early pregnancy, adverse mental stimulus, father’s drinking, history of consanguineous marriage and had a cold during early pregnancy were the main risk factors of CHD in China [9]. In this study we found that mothers’ occupation, family history with CHD, history of abortion, history of adverse pregnancy, poor early pregnancy health, fetus during pregnancy, pesticide exposure and drug exposure, and gender of children were significantly associated with the risk of CHD susceptibility. In our study, we had 8 patients with family history of CHD which included mother’s brother (1 patient), cousin (2 patients), grandfather (1 patient), mother (3 patients) or father (1patient). Among of 111 mothers in case group recalled, only 21 mothers had used of folate supplements or multiple vitamin in preconceptional period and sustained about 1month or so. Additionally, 27 mothers had used the supplements after awareness of pregnancy and sustained till the end of the first trimester of pregnancy. Unfortunately none of the mothers with a mutation of MTHFR rs1801131 CC genotypes took folate supplement or multiple vitamins during the preconceptional period. As our investigation was taken during the children with CHD were hospitalized for cure of the diseases, the mothers were confused in memory for the drug exposure because of the drugs taken happened long time ago. Moreover most of the mothers couldn’t know what medicines were used because of their education limitation. Meanwhile some mothers could only tell they were received a Chinese herbal medicine therapy which might be very complicated itself. Most of the drugs were used for fever, influenza, cough, prevent miscarriage and promote pregnancy. Similarly happened in parameters of harmful environment, the mothers recalled roughly they might be harmed by house decoration (2 cases), megatemperature (3 cases), radiation expose (2 cases), electromagnetic radiation (2 cases) and hair dye (1 case) during the early pregnancy. But they cannot describe exactly what those were and how long they were exposed in the harmful environments. Meanwhile none of the mothers in control group had such bad memories. The findings suggested that mothers’ social and fertility characteristics might affect children’s CHD incidence in some extent.

After adjustment for the above variables, we analyzed five SNPs (*MTHFR* rs1801131 and rs1801133, *MS* rs1805087, CBS rs2124459 and rs2850144) in enzymes involved in folate metabolism in 318 children and 340 mothers and studied their influence on the risk of CHD in this population. The results indicated that *MTHFR* rs1801131 was related with CHD susceptibility.

MTHFR, the major enzymes in the folate metabolism pathway, catalyzes the conversion of 5, 10 methylenetetrahydrofolate into 5-methyltetrahydrofolate. The rs1801133 polymorphism in MTHFR has been studied extensively in relation to the risk of CHD, however the results were controversial. For example, Wei Wang analyzed 29 case-control and TDT studies with MTHFR rs1801133 polymorphism and risk of CHDs and concluded that both infant and maternal MTHFR rs1801133 polymorphisms may contribute to the risk of CHDs [6]. Van Beynum IM performed a meta-analysis to find no substantial evidence of increased CHD risk in individuals with *MTHFR* rs1801133 CT and TT genotypes [5]. This probably because rs1801133 polymorphism causes the gene product to be thermolabile which causes increased plasma homocysteine concentrations but they have been shown to be prevented by periconceptional folic acid supplementation [10,11]. The prevalence of homozygosity for this polymorphism is reported to be 5% to 16% in different population. In this study we report a prevalence of about 20% in both the control and the case group in children and the mothers but no difference between them.

Van der Put and colleagues reported another common polymorphism in the *MTHFR* rs1801131 [12]. It was suggested that a combined heterozygosity for the two *MTHFR* common polymorphisms accounts for a proportion of folate-related neural tube defects. Hobbs et al examined the relation between CHD and maternal *MTHFR* polymorphisms and reported the rs1801131 allele to be transmitted less often than expected suggesting an apparent protective effect against CHD [13]. Chao et al. carried out a retrospective case-controlled study with isolated patent ductus arteriosus (PDA) patients and a control non-PDA group without CHD and reported that the *MTHFR* rs1801131 polymorphism was associated with isolated PDA in Taiwan [14]. But a meta-analysis based on 23 case-control studies by Chen et al. suggested that *MTHFR* rs1801131 is not associated with risk of CHD for Europeans [15]. In this study, after adjustment for mothers’ social and fertility characteristics, *MTHFR* rs1801131 was found related with CHD susceptibility. Zidan HE and his Colleagues also demonstrated an association of *MTHFR* rs1801131 with CHD in Egyptian children and their mothers [16]. We found that the rs1801131 (A *vs*. C) variant genotypes significantly increased their offspring CHD risk in mothers and host CHD risk in children. Hereditary analysis for the MTHFR, MS and CBS polymorphism proved that the five SNPs in mothers were significantly associated with the SNPs in their offspring. Our results suggest that the probability of occurrence of CHD in children increases when their mothers are carrying the *MTHFR* rs1801131 variant genotype.

MS catalyzes the demethylation of 5-methyl-THF to THF and the remethylation of homocysteine to methionine. A common polymorphism in the *MS* rs1805087 also seems to influence plasma homocysteine [17]. This polymorphism has been found to be associated with low levels of plasma homocysteine in a population in the Midwestern region of the USA [18]. CBS catalyzes the first irreversible step from homocysteine to cystathionine in the transsulfuration pathway. A lack of cystathionine synthase (CBS) leads to elevated plasma concentrations of homocysteine, an inherited autosomal recessive metabolic disease named homocystinuria [19]. It was reported that expressin of *CBS* was at an especially high level in the neural and cardiac systems indicating the effect of the gene to the cardiac development [20,21]. The association was not found between the *CBS* coding variants and the risk of CHD [22,23]. Zhao et al. investigated the non-coding variants in the *CBS* gene in three independent case control studies and identified a CBS promoter variant, rs2850144, which increases *CBS* gene expression and was significantly associated with reduced CHD risk in all three case-control pairs and in the combined dataset [24]. However, in this study, we did not find that the three SNPs (rs1805087, rs2850144, rs2124459) were related with the risk of CHD.

In conclusion, the polymorphism rs1801131of *MTHFR* was found related with CHD susceptibility. The higher prevalence of the rs1801131CC genotype in mothers of the affected children supports a higher risk of having a child with CHD. This implicates the genetic variants in the *MTHFR* loci may be marker SNPs for risk of susceptibility to CHD in Chinese.

## Supporting Information

S1 Table Information for Primers and Probes by TaqMan Allelic Discrimination. (PDF)
