# Supplementary material for: Study on Environmental Causes and SNPs of MTHFR, MS and CBS Genes Related to Congenital Heart Disease
Source: PLoS One. 2015 Jun 2;10(6):e0128646. doi: 10.1371/journal.pone.0128646 (PMC4452709; doi:10.1371/journal.pone.0128646)
Supplement: S1 Table — (PDF) [file pone.0128646.s001.pdf]

Supporting Table 1. Information for Primers and Probes by TaqMan Allelic Discrimination

Polymorphism	Sequence (5'-3')
rs1801133	
Primer	F :CACAAAGCGGAAGAATGTGTCA
	R : GACCTGAAGCACTTGAAGGAGAA
Probe	FAM-AAATCGGCTCCCGCA -MGB
	HEX-TGAAATCGACTCCCG-MGB
rs1801131	
Primer	F :GGAGGAGCTGCTGAAGATGTG
	R : TCTCCCGAGAGGTAAAGAACAAA
Probe	FAM-AAGACACTTTCTTCACTG -MGB
	HEX-AGACACTTGCTTCAC-MGB
rs2124459	
Primer	F :CACTTAGAGCCCCAGGAAAAGA
	R : GAAGCCTGCGGCACTCA
Probe	FAM-TCCTTGCATTTTGG -MGB
	HEX-TCCTTGCATTTCGG-MGB
rs1805087	
Primer	F :AAATCTGTTTCTACCACTTACCTTGAGA
	R : GAGGAAATCATGGAAGAATATGAAGAGA
Probe	FAM-ACTCATAATGGTCCTGTC -MGB
	HEX-ACTCATAATGGCCC-MGB
rs2850144	
Primer	F :TCCTGCCAGTGGACATTTAATTCTA
	R : TCCCCGGCTCAGGTCAGA
Probe	FAM-CAGAGACCCCGGCG -MGB
	HEX-CAGAGACCGCGGCG-MGB
